# Ultra-Wideband Double-Pentagonal Fractal Antenna for C-, X-, Ku- and K-Band Wireless Applications

**DOI:** 10.3390/mi16111237

**Published:** 2025-10-30

**Authors:** Junghyeon Kim, Taehwan Jang, Sungjoon Lim

**Affiliations:** 1 Department of Intelligent Semiconductor Engineering, Chung-Ang University, Seoul 06974, Republic of Korea; wjdgus6748@cau.ac.kr; 2 School of Electrical and Electronics Engineering, Chung-Ang University, Seoul 06974, Republic of Korea; jang101101@cau.ac.kr

**Keywords:** fractal antenna, ultra-wideband antenna, C-, X-, Ku-, K-band, double-pentagonal geometry

## Abstract

Fractal antennas employ self-similar geometries to generate scaled multiple resonances within compact structures, thereby achieving broadband performance. However, many reported designs remain constrained by narrow impedance bandwidths or demonstrate only multiband characteristics. To address these limitations, we present a novel pentagonal fractal antenna with ultra-wideband performance suitable for C, X, Ku and K-band applications. The key innovation lies in a double-pentagonal fractal configuration, created by embedding a secondary pentagonal ring within the conventional pentagonal radiator. This design significantly enhances the impedance bandwidth and enables ultra-wideband operation. The proposed antenna was validated through both electromagnetic simulations and experimental measurements. Results show a measured −10 dB impedance bandwidth of 3.84–22.4 GHz, corresponding to a fractional bandwidth of 141.5%. The antenna dimensions are only 0.384 × 0.525 × 0.01λ_0_^3^. A peak gain of 10.2 dBi was achieved, with the gain varying between 2.88 and 10.2 dBi across the operating frequency range. Owing to these characteristics, the proposed antenna is well-suited for diverse wireless communication systems, including Wi-Fi, ultra-wideband communication, 5G mid-band and emerging 6G technologies.

## 1. Introduction

Wireless communication has become an indispensable component of modern life. Smartphones, in particular, serve as gateways to a wide range of wireless systems, including Wi-Fi, mobile networks, the global positioning system (GPS), near-field communication and ultra-wideband communication [1,2,3]. In recent years, interest in satellite communication has also increased, and continued advances in communication technologies are anticipated in line with this trend [4,5]. As the diversity of communication systems expands, the frequency spectrum in use correspondingly broadens. Traditionally, the provision of these services has required multiple antennas, each dedicated to a specific frequency band. However, this approach raises device costs and can degrade performance due to mutual interference among antennas. A promising alternative is to employ a single broadband antenna capable of covering a wide frequency range [6,7,8,9,10].

Broadband antennas include spiral antennas, Vivaldi antennas, log-periodic dipole array (LPDA) antennas and planar monopole antennas. Among these, Vivaldi and LPDA antennas exhibit broadband characteristics while offering high gain, making them attractive for applications such as ground-penetrating radar [11], through-wall radar imaging [12,13] and electromagnetic interference/electromagnetic compatibility measurements [14,15]. Nevertheless, these antenna types are generally unsuitable for portable devices, as their size is relatively large and integration into compact systems is difficult. Many researchers have endeavored to overcome these limitations. For example, Moosazadeh et al. proposed a Vivaldi antenna that achieves both miniaturization and ultra-wideband performance [16]. By applying an inner-edge bending technique to a conventional antipodal Vivaldi structure, they realized an operating bandwidth from 1 to 30 GHz within a compact size of 0.32$\lambda_{0}$ × 0.46$\lambda_{0}$. However, the use of low-loss substrates increases the overall fabrication cost, which limits its feasibility for mass production. Moreover, although the directional radiation characteristics of the designed antenna make it suitable for applications such as detection of voids inside concrete specimens or radar systems, the radiation direction tends to vary depending on the user’s position or posture. Therefore, such antennas are unsuitable for portable wireless communication systems that require stable connections and uniform omni-directional radiation characteristics in all directions. In another study, H. A. Elmobarak Elobaid et al. proposed a transparent and flexible UWB antenna [17]. The proposed antenna was patterned with a conductive fabric tissue on a polydimethylsiloxane (PDMS) substrate, having a compact size of 0.293$\lambda_{0}$ × 0.366$\lambda_{0}$ and exhibiting omnidirectional radiation characteristics over 2.2–25 GHz. Such an antenna is suitable for wireless communication systems. However, the fabrication process is rather complex; the PDMS substrate must be prepared using a vacuum chamber, the transparent fabric tissue must be attached, and laser etching is required for pattern formation. In addition, due to the use of a PDMS substrate, integration with printed circuit board-based circuits is difficult. These limitations can be addressed by employing fractal antennas, which exhibit broadband characteristics, omnidirectional radiation patterns, and can be readily fabricated on PCB processes.

Fractal antennas provide an alternative approach by exploiting self-similar geometries. In miniaturized structures, such designs can generate multiple resonances, and when these resonances overlap, broadband behavior can be achieved [18,19]. These properties make fractal antennas highly suitable for applications requiring both compact size and wide bandwidth [20,21,22]. Nonetheless, several challenges remain. Achieving stable broadband performance requires multiple resonances to occur at appropriate frequencies and to overlap effectively. Resonances arise from the equivalent inductive and capacitive elements formed by the antenna geometry. Modifying the geometry to create resonance at a specific frequency inevitably influences other resonances, which complicates efforts to achieve broadband stability. Consequently, most prior studies have concentrated either on obtaining broadband performance within limited frequency ranges or on developing multiband characteristics [23,24,25,26,27,28,29,30]. For example, Marzouk et al. proposed a wideband fractal antenna for WiMAX, WLAN, C-band and X-band applications [31]. By applying iterative geometric transformations to a circular patch, they optimized the impedance bandwidth and achieved dual-wideband performance, with −10 dB bandwidths of 2.26–4.1 GHz and 6.1–10 GHz. Although effective for several applications, this design remains less suitable for some C-band operations. In another study, Nejdi et al. proposed a wideband fractal antenna optimized by iterative modifications of a circular geometry [32]. Their design achieved an impedance bandwidth from 2.83 to 10.16 GHz, making it applicable to certain X-band systems but limited at higher frequencies.

In this work, we propose a pentagonal fractal antenna with a substantially wider impedance bandwidth. The design employs a double-pentagonal fractal structure, formed by integrating an inner pentagonal ring within the conventional geometry, which significantly enhances the bandwidth. The antenna was fabricated using PCB processes and validated through simulation and measurement. Results show an impedance bandwidth of 3.36–23.56 GHz in simulation and 3.84–22.44 GHz in measurement (|S_11_| ≤ −10 dB). These correspond to fractional bandwidths of 150% and 141.5%, respectively. Remarkably, this performance was achieved with a compact antenna size of only 0.384 × 0.525 × 0.01λ_0_^3^ (at the lowest operating frequency), which is comparable with related work. Far-field radiation patterns were also measured and found to agree well with simulated results.

The remainder of this paper is organized as follows. Section 2 details the design procedure of the proposed antenna, its simulated results and the operating principle. Section 3 presents the measurement results of the fabricated prototype. Section 4 provides a comparative discussion with previous studies. Finally, Section 5 concludes the paper.

## 2. Proposed Wideband Fractal Antenna Design and Simulation

### 2.1. Fractal Antenna Design Process

**Figure 1:**
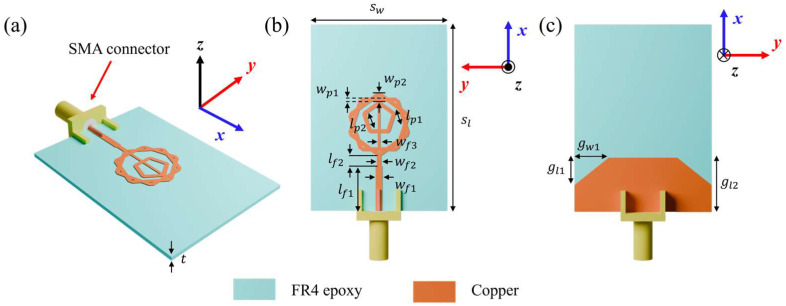
Proposed antenna design: (**a**) bird’s-eye view, (**b**) top view and (**c**) bottom view.

**Figure 2:**
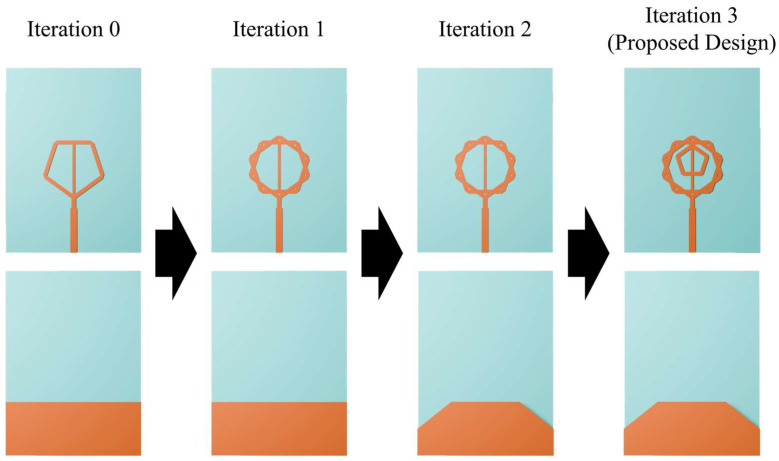
Iterative design process of the proposed pentagonal fractal antenna.

Figure 1a–c presents the bird’s-eye, top and bottom views of the proposed ultra-wideband fractal antenna. An FR4-epoxy substrate with a dielectric constant of 4.4 and loss tangent of 0.018 was used. The antenna employs a double-pentagonal configuration in which an inner pentagonal ring is embedded within the conventional fractal radiator. A ground-plane truncation technique was adopted to improve impedance matching across a wide frequency range. For practical fabrication and characterization, an SMA (SubMiniature A) connector was incorporated. The optimized antenna parameters are: $s_{w}$ = 30 mm, $s_{l}$ = 41 mm, $w_{p1}$ = 0.9 mm, $w_{p2}$ = 1.9 mm, $l_{p1}$ = 3.26 mm, $l_{p2}$ = 2.9 mm, $w_{f1}$ = 1.5 mm, $w_{f2}$ = 1 mm, $w_{f3}$ = 0.6 mm, $l_{f1}$ = 9.9 mm, $l_{f2}$ = 2.2 mm, $g_{w1}$ = 7.5 mm, $g_{l1}$ = 6 mm, $g_{w2}$ = 11.9 mm and t = 0.9 mm. Figure 2 illustrates the iterative evolution of the design from a simple pentagonal radiator to the final double-pentagonal fractal antenna. This study aimed to achieve a reflection coefficient bandwidth operating below −10 dB in the frequency bands used for UWB communications (3.1–10.6 GHz) and next-generation 6G communications (expected to utilize the 14.8–15.35 GHz band). To meet these conditions, we first defined adjustable design parameters in the simulation tool for each iteration step. We performed simulations while modifying parameter values, and after securing the widest bandwidth, we proceeded to the next step of design modification. Specifically, in Iteration 0, optimization was performed by adjusting the size of the pentagon and the length and width of the signal line. However, the reflection coefficients still exceeded −10 dB in the 5–9 GHz and 16 GHz bands. Therefore, we modified the design using the self-similar fractal generation. Applying the self-similar fractal generation to the antenna improves impedance matching and consequently enables a wide bandwidth [33,34]. The Iteration 1 structure was modified by merging the pentagonal design from Iteration 0 with an identical pentagonal design rotated by 36°. The modified Iteration 1 structure gains additional capacitance from the newly formed slots, which helped improve bandwidth. However, impedance matching was still insufficient in certain frequency ranges, particularly at lower frequencies. Therefore, the ground truncation technique was applied to improve impedance matching in the low-frequency band. In Iteration 2, the parameters associated with the ground plane cutting technique were optimized to achieve impedance matching in the low-frequency band. However, impedance matching was still not satisfactory around the 10 GHz and 20 GHz bands. Finally, by applying a self-similar fractal generation rule, in which an additional pentagonal element was introduced at the center of the antenna, we achieved an optimal impedance bandwidth. The mechanism behind these results can be explained by the theory of printed monopole antennas. Specifically, the proposed fractal antenna is a type of printed monopole antenna, and its low-frequency resonance $f_{L}$ can be estimated using the formula for an equivalent cylindrical monopole antenna, as expressed in [35]:

(1) $$f_{L}=\frac{c}{\lambda }=\frac{7.2}{\left(L+r+p\right)}\;GHz,$$

where L is the height of the planar monopole antenna (in cm), r is the effective radius of the equivalent cylindrical monopole antenna (in cm), and p is the length of the 50 Ω feed line (in cm). The proposed microstrip line-based printed monopole antenna includes a dielectric layer on one side, which increases the effective electrical dimensions and lowers the low-frequency resonance. The equation considering the effective permittivity is given as [36]:

(2) $$f_{L}=\frac{7.2}{\left\{\left(L+r+g\right)\;\times \sqrt{\varepsilon_{eff}}\right\}}\;GHz,$$

where g is the gap between the fractal-structured radiator and the ground plane. The value of g is determined by ${(l}_{f1}+\;l_{f2})-\;g_{l2}$. The effective permittivity can be calculated using the following equation:

(3) $$\varepsilon_{eff}=\frac{\varepsilon_{r}+1}{2}+\;\frac{\varepsilon_{r}-1}{2}\;{\left(1+12\frac{h}{W}\right)}^{-\frac{1}{2}},\;$$

where W is the width of the microstrip line and h is the thickness of the substrate. The equation indicates that the resonant frequency varies with the radius of the fractal-structured radiator. The proposed double-pentagonal fractal antenna can be regarded as a combination of two monopole antennas of different sizes. Therefore, by inserting an additional pentagonal element that generates resonance in the impedance-mismatched frequency region, we were able to achieve an optimal impedance bandwidth. This stepwise procedure demonstrates how the simple pentagonal geometry is transformed to achieve ultra-wideband behavior.

### 2.2. Antenna Simulation

**Figure 3:**
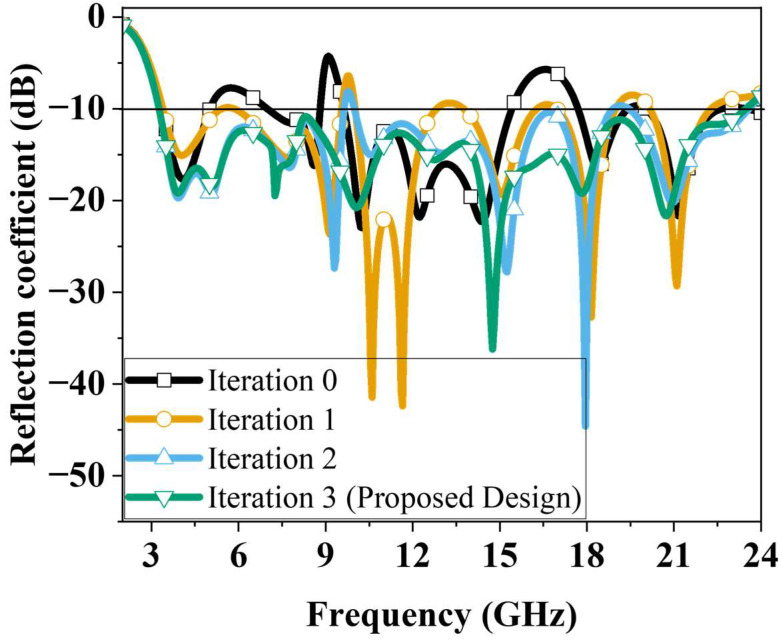
Simulated reflection coefficients for different iterations.

Figure 3 presents the simulated reflection coefficients obtained from the iterative design process. Simulations were performed using ANSYS high-frequency structure simulator (HFSS 2016.02 version). For the 0th-iteration structure, the reflection coefficient remains above −10 dB over most of the 3–24 GHz range, showing particularly strong impedance mismatching around 10 and 16 GHz. In the 1st-iteration structure, the mismatch around 16 GHz is improved; however, many frequency regions still exhibit reflection coefficients slightly above −10 dB, and the mismatch near 10 GHz remains unresolved. In the 2nd iteration, the application of the ground truncation technique enhances impedance matching in the low-frequency region, but the mismatch around 10 GHz still persists. Finally, the proposed antenna achieves stable impedance characteristics across all previously mismatched frequency bands, maintaining reflection coefficients below −10 dB throughout 3.36–23.56 GHz, corresponding to a bandwidth of 20.2 GHz.

**Figure 4:**
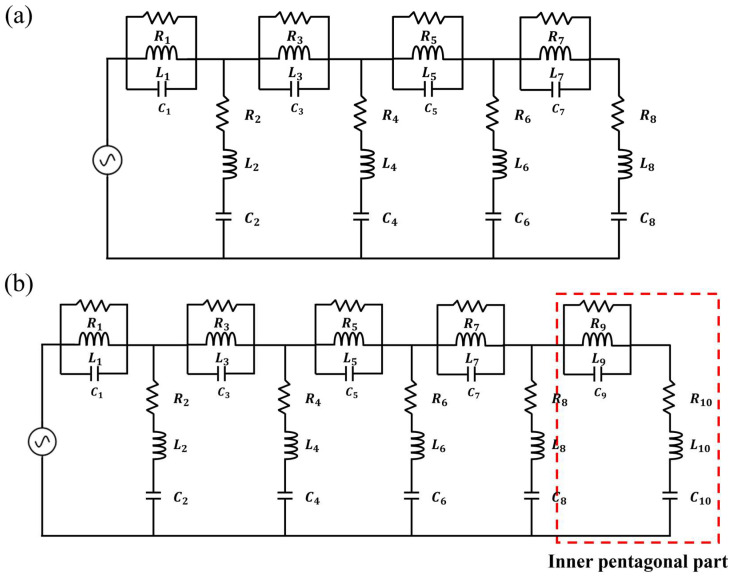
Equivalent circuit model: (**a**) Iterations 0–2 structures and (**b**) Iteration 3 (the proposed antenna) structure.

**Figure 5:**
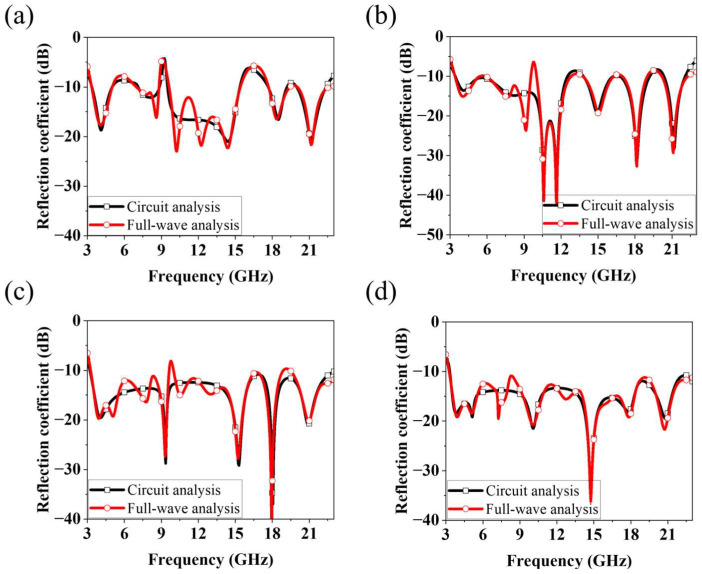
Comparison of reflection coefficients from full-wave analysis and circuit analysis: (**a**) Iterations 0, (**b**) Iteration 1, (**c**) Iteration 2 and (**d**) Iteration 3 (the proposed antenna) structures.

Figure 4 illustrates the equivalent circuit models from Iteration 0 to Iteration 3. As shown in Figure 4a, the structures from Iteration 0 to 2 can be equivalently modeled using eight series–parallel RLC circuits. The main difference among these iterations lies in the variation in the RLC values. From Iteration 0 to 2, the pattern geometry was modified and the ground truncation technique was applied; these changes did not add new circuits but rather adjusted the existing LC values to achieve impedance matching. As shown in Figure 4b, the Iteration 3 structure can be represented by ten series–parallel RLC circuits, where two additional circuits are generated by the inserted inner pentagonal ring pattern. These newly created circuits introduce additional resonances and compensate for the impedance mismatches that remained in the Iteration 2 structure. Figure 5 presents a comparison between the circuit analysis results and the full-wave analysis results. The circuit analysis was performed using the Keysight advanced design system tool. Figure 5a–c show the comparison results for the structures from Iteration 0 to Iteration 2. It can be observed that the overall trends of the two analyses are in good agreement. All three structures can be equivalently modeled using eight series–parallel RLC circuits, each having different RLC values. This indicates that the modifications applied from Iteration 0 to Iteration 2 improved the impedance matching by varying the RLC parameters corresponding to the pattern geometry changes. Figure 5d shows the comparison between the circuit and full-wave results for the proposed ultra-wideband fractal antenna (Iteration 3). This structure can be represented by ten series–parallel RLC circuits, including two additional circuits generated by the inserted inner pentagonal ring pattern. The two sets of results exhibit a highly similar trend, demonstrating that the inner pentagonal ring of the proposed antenna introduces new resonances that compensate for the impedance mismatches observed in the Iteration 2 structure, thereby achieving optimal ultra-wideband impedance matching. The RLC values used for each iteration in the circuit analysis are summarized in Table 1.

**Figure 6:**
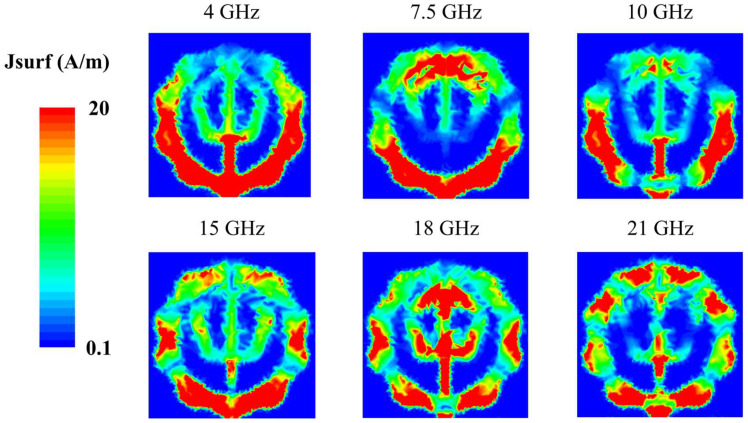
Simulated surface current distributions at 4, 7.5, 10, 15, 18 and 21 GHz.

Figure 6 shows the simulated surface current distributions of the proposed antenna at representative resonant frequencies (4, 7.5, 10, 15, 18 and 21 GHz). The six frequencies were selected because they exhibited distinct resonance behavior in the reflection coefficient results. At 4 GHz, currents are concentrated near the feeding region and extend across both the outer and inner pentagonal structures. At 7.5 GHz, current flows shift predominantly to the upper and lower edges of the outer pentagon, with a similar distribution at 10 GHz, albeit with weaker feed intensity. At 15 GHz, strong currents are observed in the outer pentagon near the feed point, while at 18 GHz they are primarily concentrated on the inner pentagon. At 21 GHz, significant currents appear across both the inner and outer pentagonal geometries. The proposed antenna shows a tendency for the region of strong surface current to become shorter as the frequency increases. This result indicates that the antenna’s resonance behavior is mainly governed by the effective current path length. Furthermore, it is confirmed that the double-pentagonal structure contributes to the wideband characteristics of the antenna.

**Figure 7:**
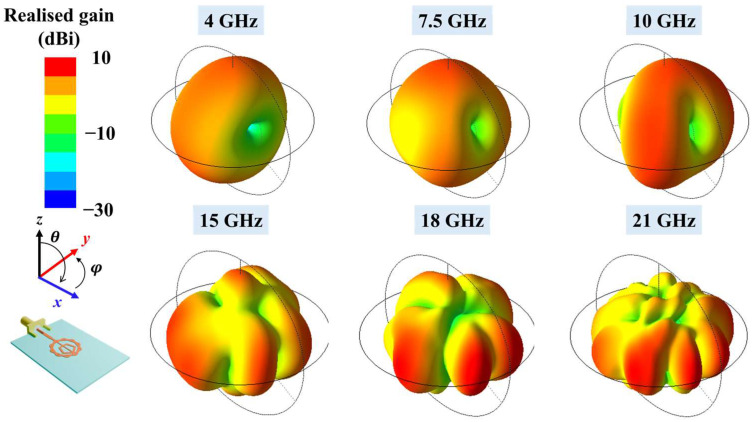
Simulated 3D radiation patterns of the proposed fractal antenna at 4, 7.5, 10, 15, 18 and 21 GHz.

Figure 7 illustrates the simulated 3D radiation patterns at the same frequencies. At 4 GHz and 7.5 GHz, the antenna exhibits dipole-like, near-omnidirectional radiation. From 10 GHz upwards, the main beam begins to tilt, and from 15 GHz onwards, multi-beam radiation patterns are observed. The number of beams increases with frequency. At 21 GHz, the broadside gain decreases substantially, while the field intensity in the end-fire direction increases.

**Figure 8:**
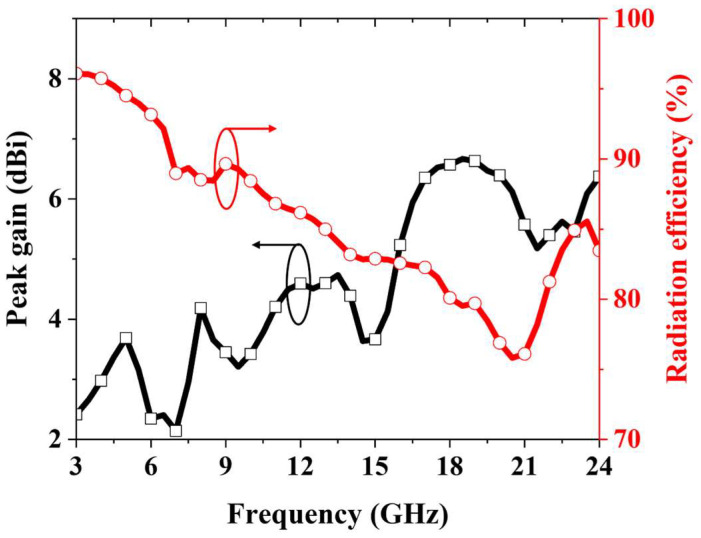
Simulated peak gain and radiation efficiency of the proposed fractal antenna.

Figure 8 shows the simulated peak gain and radiation efficiency across the operating band. The peak gain increases gradually with frequency, from 2.15 dBi at the lower band to 6.6 dBi at the upper band. Radiation efficiency is 96.1% at 3 GHz and decreases progressively with frequency, reaching a minimum of 75.8% at 20.5 GHz, before rising again at higher frequencies. This decline in radiation efficiency is primarily attributed to the increased dielectric losses of the FR4 substrate and elevated conductor losses at higher frequencies.

### 2.3. Parametric Study

**Figure 9:**
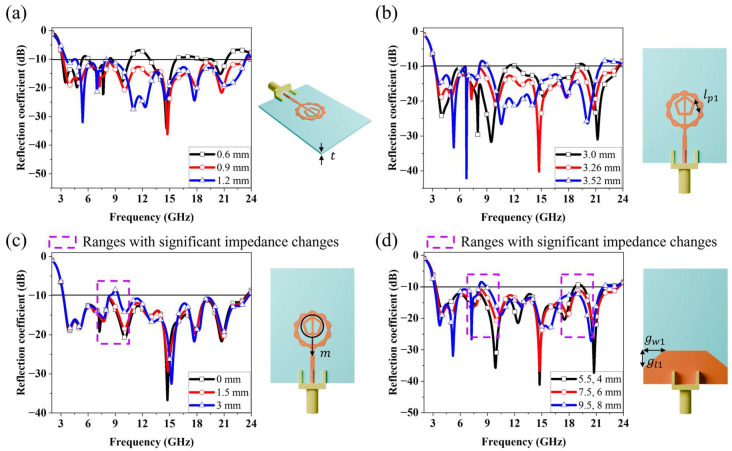
Simulated reflection coefficient results for different values of (**a**) t, (**b**) $l_{p1}$, (**c**) m and (**d**) $g_{w1}$, $g_{l1}$.

A parametric study was conducted to evaluate the influence of key design parameters on the antenna’s impedance performance. Figure 9a illustrates the effect of varying the substrate thickness t from 0.6 to 1.2 mm. When t is 0.6 mm, it can be observed that most frequency bands exhibit reflection coefficients higher than −10 dB. As the thickness increases, impedance matching tends to become more stable; however, the starting point of the operating frequency gradually shifts toward higher frequencies. Therefore, the dielectric thickness t was set to 0.9 mm to obtain optimal results. Figure 9b shows the results when the length $l_{p1}$ of one side of the outer polygon was varied from 3.00 mm to 3.52 mm in increments of 0.26 mm. The results indicate that at 3.00 mm, regions appear where the reflection coefficient exceeds −10 dB in the 12 GHz and 19 GHz bands. At 3.26 mm, all bands exhibit reflection coefficients below −10 dB, indicating good impedance matching; however, at 3.52 mm, impedance matching deteriorates in the 10 GHz band. In addition, the resonance point observed near 6 GHz shifts to higher frequencies, and matching degradation occurs at the edges of the operating frequency range. These results clearly show that the reflection coefficient is highly sensitive to variations in $l_{p1}$, and the optimal value was determined to be 3.26 mm. Figure 9c shows the reflection coefficient results when the inner pentagonal ring is moved toward the feed direction by a distance m. The parameters were redefined and re-simulated because, although the reflection coefficient differed significantly depending on the presence of the inner pentagonal ring, the variation with its size was negligible. The simulation results indicate that as the pentagonal ring moves toward the feed direction, impedance matching deteriorates in the 9–13 GHz band. This phenomenon occurs because the resonance near 10 GHz becomes weaker. This phenomenon occurs because the resonance in the 10 GHz band becomes weaker, which is caused by the pattern shifting into the region where a strong field is formed between the outer polygon and the inner pentagon, resulting in significant changes in current formation. Figure 9d shows the influence of the ground-truncation dimensions, with $g_{w1}$ and $g_{l1}$ varied from 5.5 and 4 mm to 9.5 and 8 mm, respectively. When $g_{w1}$ and $g_{l1}$ are 5.5 mm and 4.0 mm, respectively, the results show degraded impedance matching in the 20 GHz band and at the upper end of the operating frequency range. Although impedance in the previously mismatched frequency bands tends to improve as $g_{w1}$ and $g_{l1}$ increase, impedance matching deteriorates in the 9 GHz band. Furthermore, the improvement in impedance matching near the edges of the operating frequency range also becomes unstable. These results demonstrate the critical importance of determining the optimal parameter values when applying the ground truncation technique.

## 3. Fabrication and Measurement

**Figure 10:**
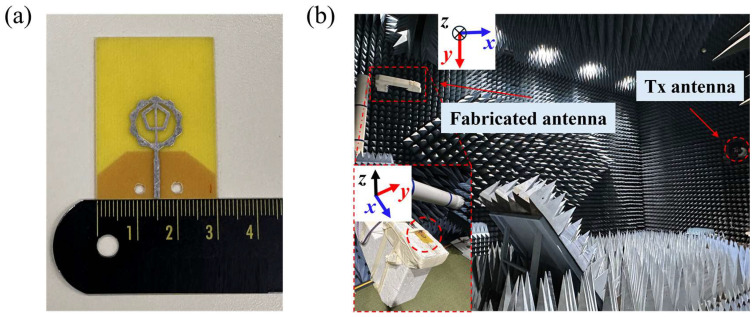
(**a**) Fabricated fractal antenna prototype and (**b**) far-field measurement setup.

**Figure 11:**
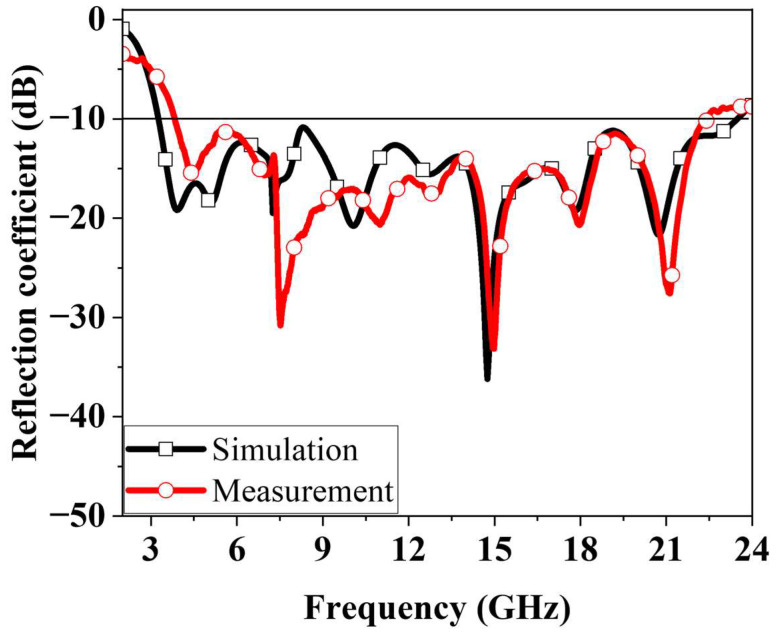
Simulated and measured reflection coefficients of the proposed fractal antenna.

The optimized antenna was fabricated using standard PCB manufacturing processes. A photograph of the fabricated prototype is shown in Figure 10a. The reflection coefficient was measured using a Keysight N5227B performance network analyser (Santa Rosa, CA, USA). Far-field radiation characteristics were evaluated using the measurement setup shown in Figure 10b. Figure 11 compares the simulated and measured reflection coefficients of the antenna. The −10 dB impedance bandwidth obtained from simulation was 3.36–23.56 GHz, whereas the measured result was 3.84–22.44 GHz. The close agreement between simulation and measurement results show good agreement.

**Figure 12:**
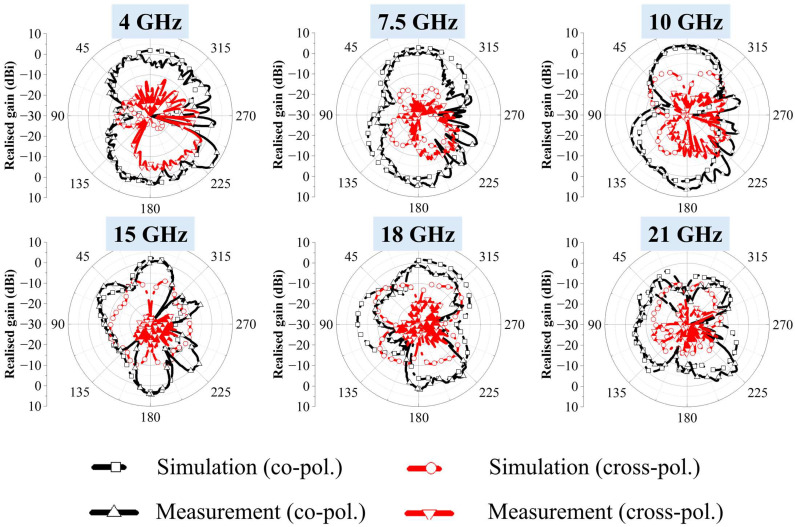
Simulated and measured radiation patterns of the proposed fractal antenna at 4, 7.5, 10, 15, 18 and 21 GHz (φ = 0°).

Figure 12 presents the simulated and measured two-dimensional radiation patterns in the *φ* = 0° plane. Across all frequencies, the measured patterns are broadly consistent with simulation, although noticeable ripples appear in the measurements. This discrepancy is mainly attributed to the use of an end-launch connector. The polytetrafluoroethylene dielectric section of the connector was too short, resulting in a short-circuit when attached directly to the antenna. To mitigate this, a small gap was introduced between the connector and the antenna, which introduced additional capacitance and consequently affected the measured patterns. Nevertheless, the cross-polarized components consistently remain lower than the co-polarized main beam at all measured frequencies. The measured radiation patterns exhibit a figure-eight shape in the *φ* = 0° plane, which is typical of an omni-directional antenna. This behavior arises because the proposed ultra-wideband fractal antenna is a printed monopole type, and monopole antennas are well known for their omni-directional radiation characteristics. The antenna maintains an omni-directional pattern up to approximately 10 GHz; however, the pattern becomes distorted above 15 GHz. This distortion occurs as the influence of cross-polarization increases with frequency, leading to pattern asymmetry. In addition, the excitation of higher-order modes at higher frequencies causes the surface current distribution to become more complex, which further degrades the radiation pattern [37,38].

**Figure 13:**
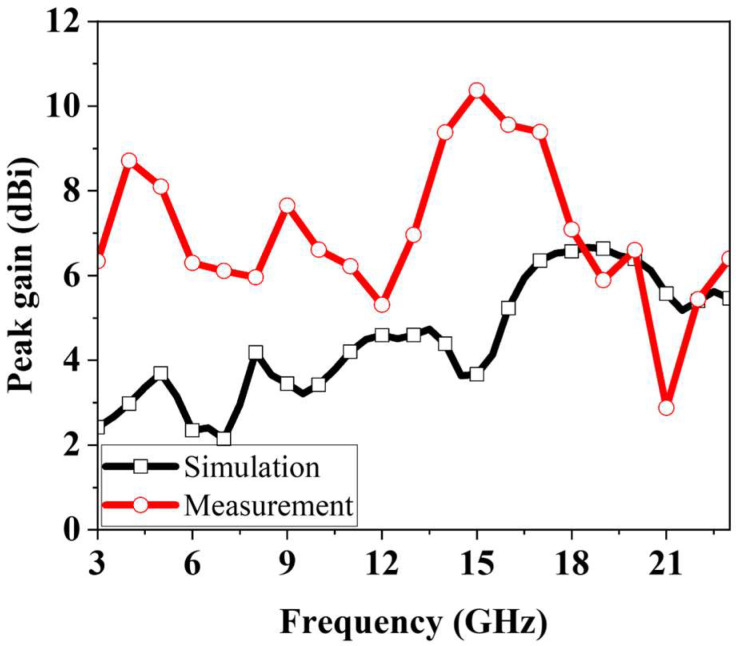
Measured peak gain of the proposed fractal antenna.

Figure 13 shows the measured peak gain of the fabricated antenna. Compared with simulation, the measurements indicate higher gain across much of the operating band. This difference is primarily due to the ripples in the measured radiation patterns, which contributed to artificially elevated peak gain values at certain frequencies. The measured gain varies between 2.88 and 10.2 dBi across the band.

## 4. Discussion

Table 2 provides a comparison between the proposed broadband fractal antenna and previously reported fractal antenna designs. Most earlier studies have concentrated on dual-band operation. For example, the design in [31] operates over 2.26–9.82 GHz but exhibits a reflection coefficient above −10 dB in the 4.1–6 GHz range, rendering it unsuitable for certain C-band applications. Efforts to develop broadband fractal antennas have also been reported. In [32], a broadband fractal antenna was presented with an operating range of 2.83–10.16 GHz. While effective, its coverage is limited to part of the C-band through to the X-band, restricting its applicability in wider frequency domains. In contrast, the proposed design achieves continuous ultra-wideband operation from 3.84 to 22.4 GHz. This wide coverage supports applications across the S-, X-, Ku- and K-bands, thereby accommodating a broad spectrum of wireless communication systems, including Wi-Fi, ultra-wideband communications and 5G mid-band services. Importantly, the antenna also covers candidate 6G frequency ranges, such as 7.125–8.4 GHz and 14.8–15.35 GHz, which are under active consideration for next-generation mobile communication systems. However, the proposed antenna exhibits radiation pattern distortion above 15 GHz, resulting in pronounced multi-lobe and back-radiation characteristics. This behavior limits its suitability for directional communication systems such as satellite links and radar operating in the Ku- and K-bands. Nevertheless, it can be effectively utilized in applications that require omnidirectional radiation characteristics, such as point-to-multipoint communication and wireless sensing systems [39,40,41].

In addition, the proposed antenna is larger in size compared with previously reported designs. This increase in size results from the inherent trade-off between antenna dimensions and operating bandwidth. In order to achieve a stable and wide bandwidth in fractal antennas, multiple resonances must be generated. Although these multiple resonances are produced by the self-similar geometric patterns of fractal antennas, as demonstrated in this study, certain frequency regions still exhibit impedance mismatching. To compensate for these mismatched regions, additional conductive patterns corresponding to the specific resonance frequencies are required. The introduction of new patterns for improved impedance matching necessitates additional non-conductive areas, which consequently leads to an increase in the overall antenna size.

## 5. Conclusions

This study proposes a novel ultra-wideband double-pentagonal fractal antenna. To address the limitations of conventional fractal antennas, which are typically narrowband or multi-band, a new design approach was proposed. Ultra-wideband performance was achieved by integrating additional pentagonal loops into the fractal geometry and employing a ground-plane truncation technique. The resulting antenna operates across 3.84–22.4 GHz while maintaining compact dimensions of 0.384 × 0.525 × 0.01λ_0_^3^, comparable to previous designs. Fabrication is straightforward using standard PCB processes on a low-cost FR4 substrate, facilitating potential mass production. The proposed antenna supports a broad spectrum of wireless communication applications, spanning the S-, X-, Ku- and K-bands, and includes candidate frequency ranges for next-generation 6G communications.

## Figures and Tables

**Table 1:** Component values of each iteration structure.

Element	Values (Ω, Iteration 0/1/2/3)	Element	Values (nH, Iteration 0/1/2/3)	Element	Values (pF, Iteration 0/1/2/3)
$R_{1}$	54/43.1/0.0226/11.03	$L_{1}$	4.97/5.75/1.242/198	$C_{1}$	0.214/0.036/0.07/8.4
$R_{2}$	47/32/30.1/48.6	$L_{2}$	3.1/4.1/0.018/18.75	$C_{2}$	0.02/0.0151/1.54/0.13
$R_{3}$	1.6/0.06/0.92/2.33	$L_{3}$	0.28/0.263/0.233/0.74	$C_{3}$	1.3/0.653/0.65/0.067
$R_{4}$	145/30.8/30.5/11	$L_{4}$	0.03722/0.45/1.97/0.02	$C_{4}$	7.95/0.83/0.119/13.1
$R_{5}$	102/32.9/17/20	$L_{5}$	0.047/0.05/0.012/0.012	$C_{5}$	2.16/2.23/23.9/4.76
$R_{6}$	71.1/80/43/32.5	$L_{6}$	2.1/0.096/0.0118/135.2	$C_{6}$	0.185/1.95/4.9/0.084
$R_{7}$	312/80/865/6	$L_{7}$	0.085/1.651/0.82/0.014	$C_{7}$	1.08/0.434/1.14/70
$R_{8}$	0.068/9.4/1.08/0.011	$L_{8}$	58.2/98.6/33.7/0.15	$C_{8}$	9.54/40.9/9.93/1.5
$R_{9}$	-/-/-/18.5	$L_{9}$	-/-/-/0.0106	$C_{9}$	-/-/-/8
$R_{10}$	-/-/-/0.00025	$L_{10}$	-/-/-/0.064	$C_{10}$	-/-/-/0.64

**Table 2:** Comparison of the proposed ultra-wideband fractal antenna with previously reported designs.

Ref.	Reflection Coefficient Bandwidth (GHz)	FBW ^1^ (%)	Peak Gain (dBi)	Antenna Size $\left({\lambda_{0}}^{3}\right)$
[24]	1.6–1.96/3.16–3.55	20.2/11.6	2.09	0.266 × 0.266 × 0.008
[26]	1.9–2.5/5–6	27.3/18.2	11.8	N/A
[27]	1.63–1.88/4.5–8.5	14.2/30.7	5.05	0.147 × 0.272 × 0.008
[28]	3.2–7.5	80.4	6.8	0.362 × 0.319 × 0.017
[31]	2.26–4.1/6–9.82	57.8/48.3	9	0.3 × 0.255 × N/A
[32]	2.83–10.16	112.8	6.25	0.377 × 0.231 × 0.015
This work	3.84–22.4	141.5	10.2	0.384 × 0.525 × 0.01

^1^ The fractional bandwidth (FBW) is defined as FBW = 2 (*f_H_* − *f_L_*)/(*f_H_* + *f_L_*), where *f_H_* and *f_L_* are the upper and lower bounds of the −10 dB impedance bandwidth, respectively.

## Data Availability

The data presented in this study are available on request from the corresponding author.
